# Corrigendum to: A continuous murmur in an elderly woman

**DOI:** 10.1093/ehjcr/ytaa438

**Published:** 2020-11-17

[Eur Hear J Case Report 2020; doi:10.1093/ehjcr/ytaa245].

In the originally published version of this manuscript, Corresponding author’s telephone number was incorrect. This has now been corrected online.

